# Supplementary material for: The genus Serratia revisited by genomics
Source: Nat Commun. 2022 Sep 3;13:5195. doi: 10.1038/s41467-022-32929-2 (PMC9440931; doi:10.1038/s41467-022-32929-2)
Supplement: Supplementary file 1 — Supplementary Information [file 41467_2022_32929_MOESM1_ESM.pdf]

Supplementary Information

The genus *Serratia* revisited by genomics

Williams *et al*.

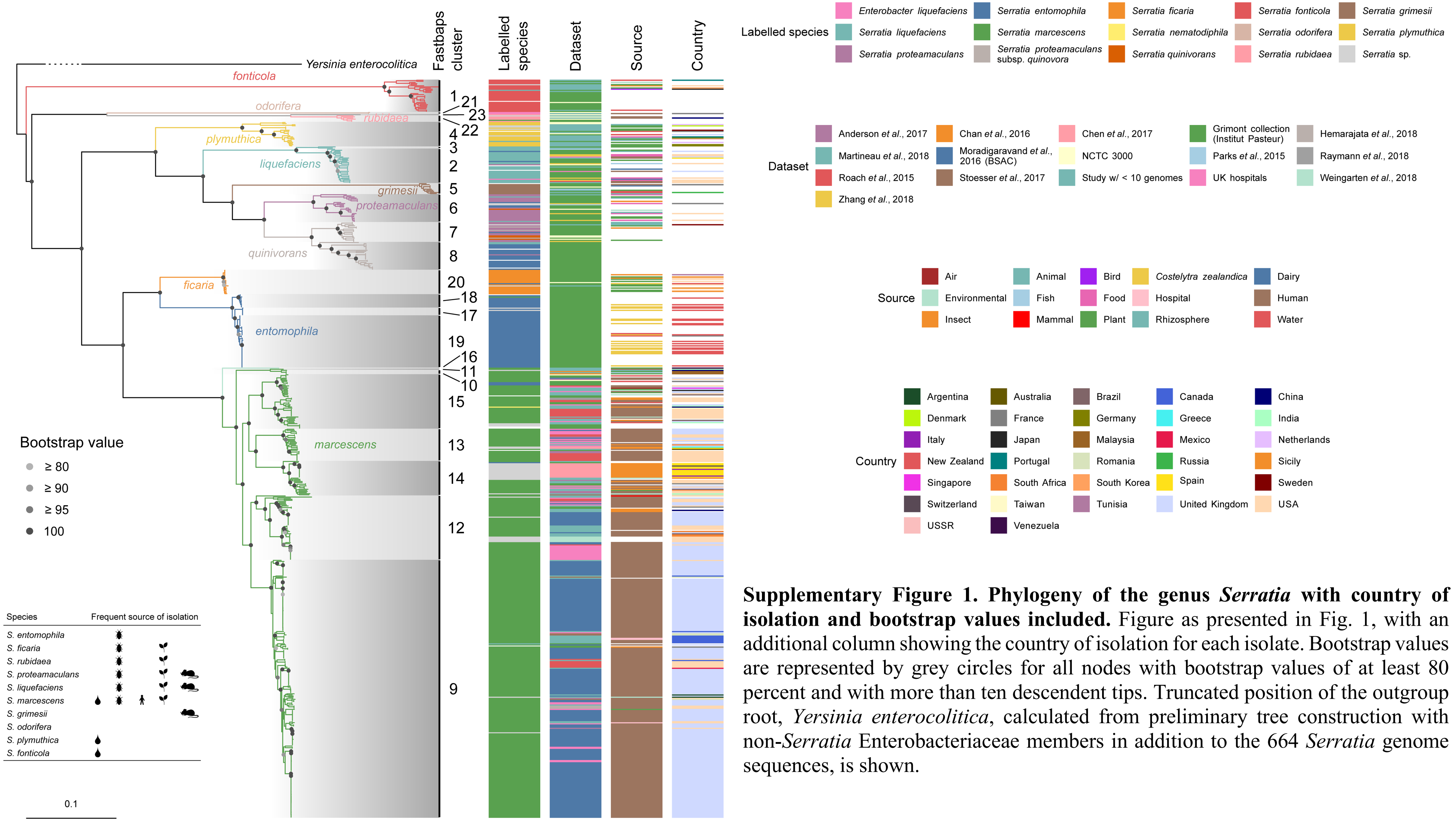

Supplementary Figure 1. Phylogeny of the genus *Serratia* with country of isolation and bootstrap values included. Figure as presented in Fig. 1, with an additional column showing the country of isolation for each isolate. Bootstrap values are represented by grey circles for all nodes with bootstrap values of at least 80 percent and with more than ten descendent tips. Truncated position of the outgroup root, *Yersinia enterocolitica*, calculated from preliminary tree construction with non-*Serratia* Enterobacteriaceae members in addition to the 664 *Serratia* genome sequences, is shown.

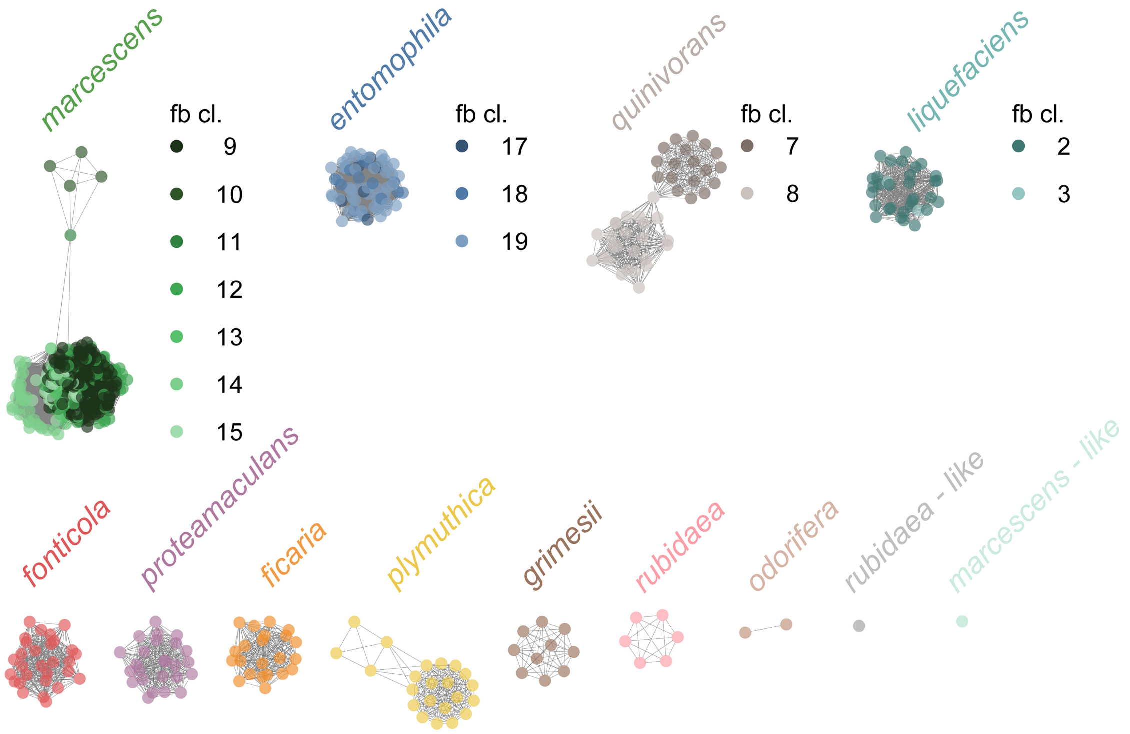

Supplementary Figure 2. ANI clusters of the *Serratia* genus dataset. Undirected network of pairwise average nucleotide identity (ANI) for all genome sequences in the *Serratia* genus dataset, determined using fastANI, using a cutoff of 95%. Nodes are coloured according to the species attributed to each cluster. For clusters comprising more than one lineage, as determined using FastBaps, nodes are shaded according to each lineage and numbered by the FastBaps cluster (fb cl.).

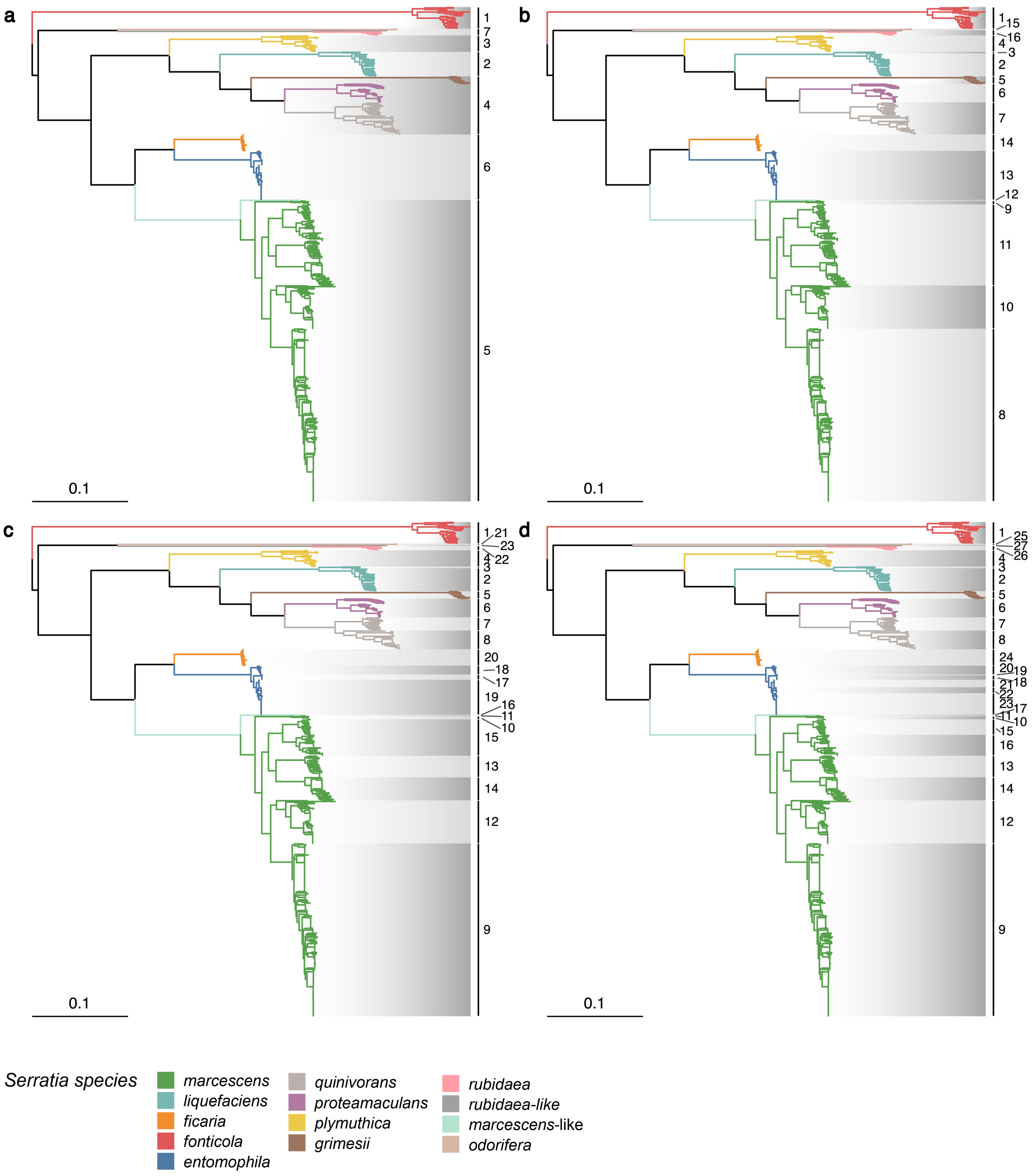

Supplementary Figure 3. FastBaps clustering levels 1-4 across *Serratia*. Phylogenetic trees shown as in Fig. 1. Clades shaded according to FastBaps levels: (a) 1 (b) 2 (c) 3 (d) 4.

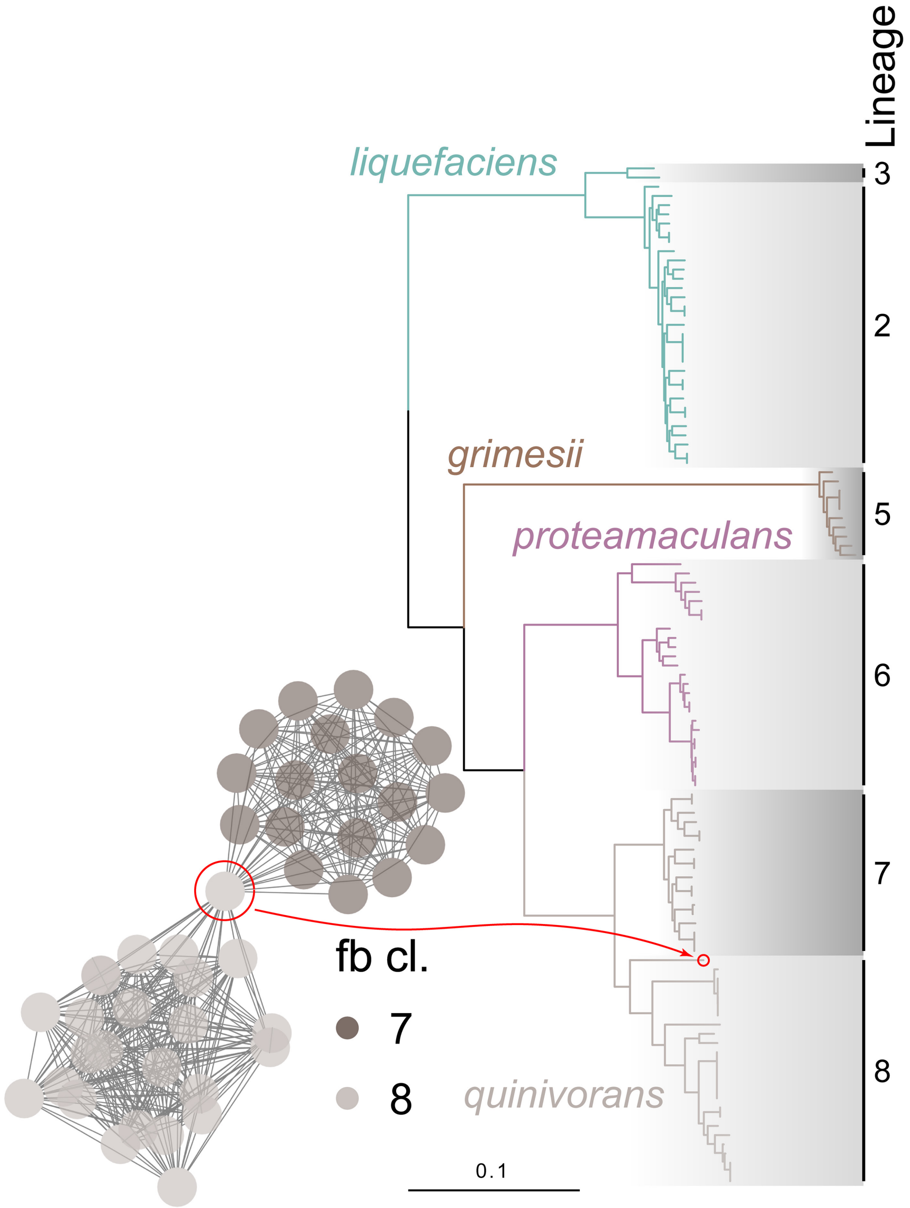

Supplementary Figure 4. *Serratia quinivorans* comprises two lineages, linked into a single ANI group connected by a single genome. ANI network and FastBaps clustering for *quinivorans* reproduced from Supplementary Fig. 3 (fb cl., FastBaps cluster).

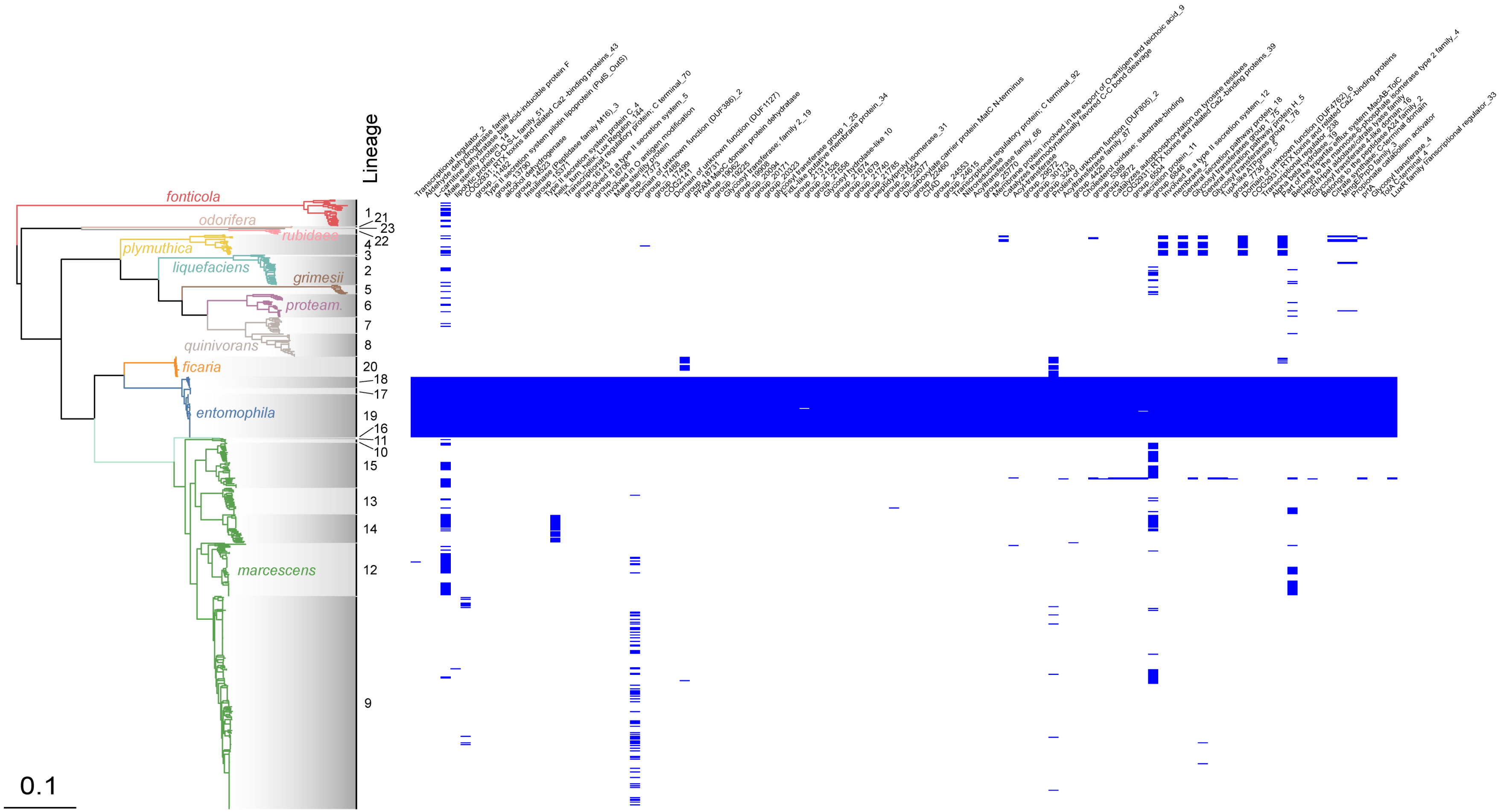

Supplementary Figure 5. Distribution of genes core to *S. entomophila* (FastBaps L17-19). Intersection of genes which are only core to lineages 17-19, extracted and plotted against the phylogenetic tree of *Serratia*. Intersection is highlighted on Fig. 2b.

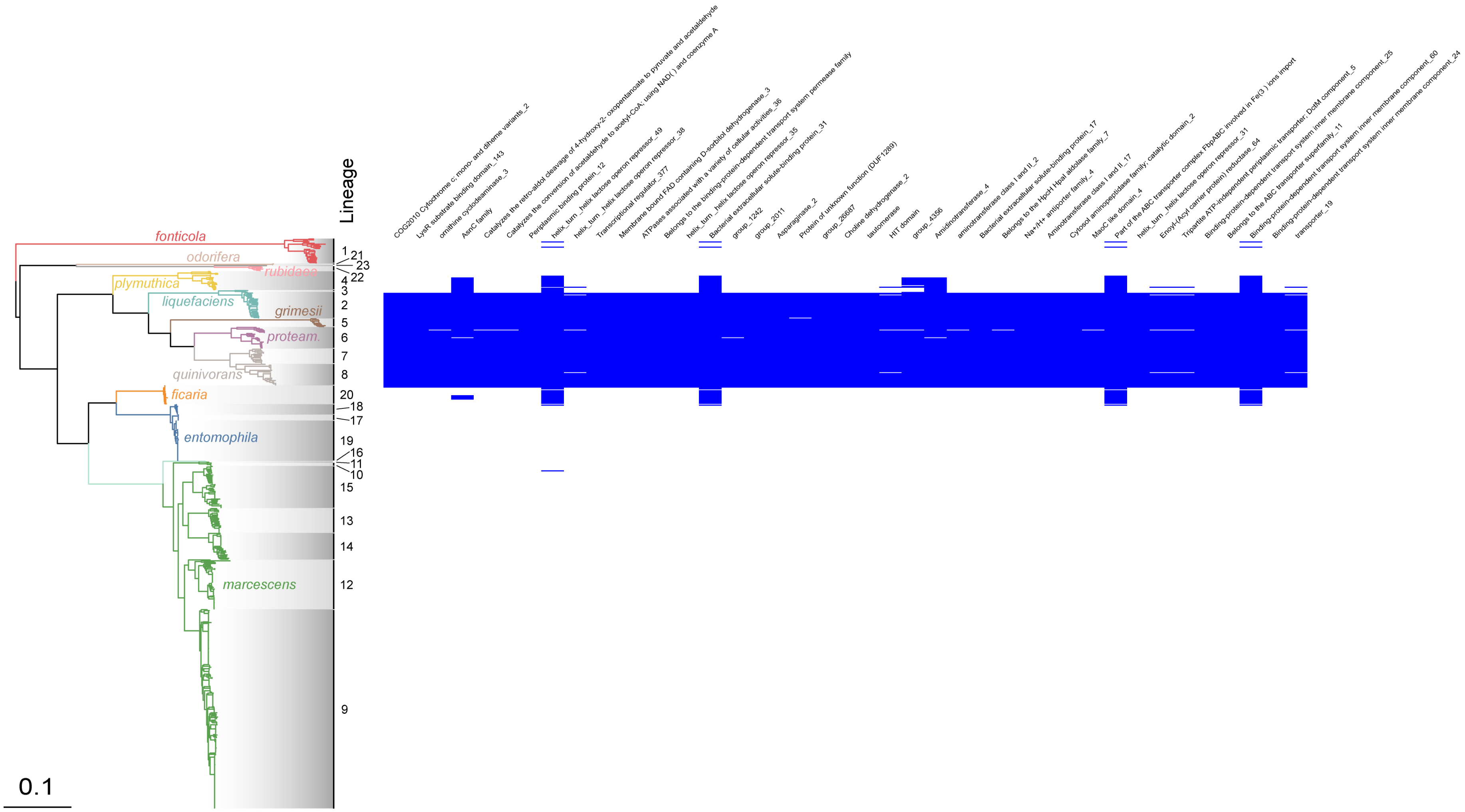

Supplementary Figure 6. Distribution of genes core to the *liquefaciens* complex (*S. liquefaciens, grimesii, proteamaculans, quinivorans*; FastBaps L2, 3, 5-8). Intersection of genes which are only core to lineages 2, 3, 5-8, extracted and plotted against the phylogenetic tree of *Serratia*. Intersection is highlighted on Fig. 2b.

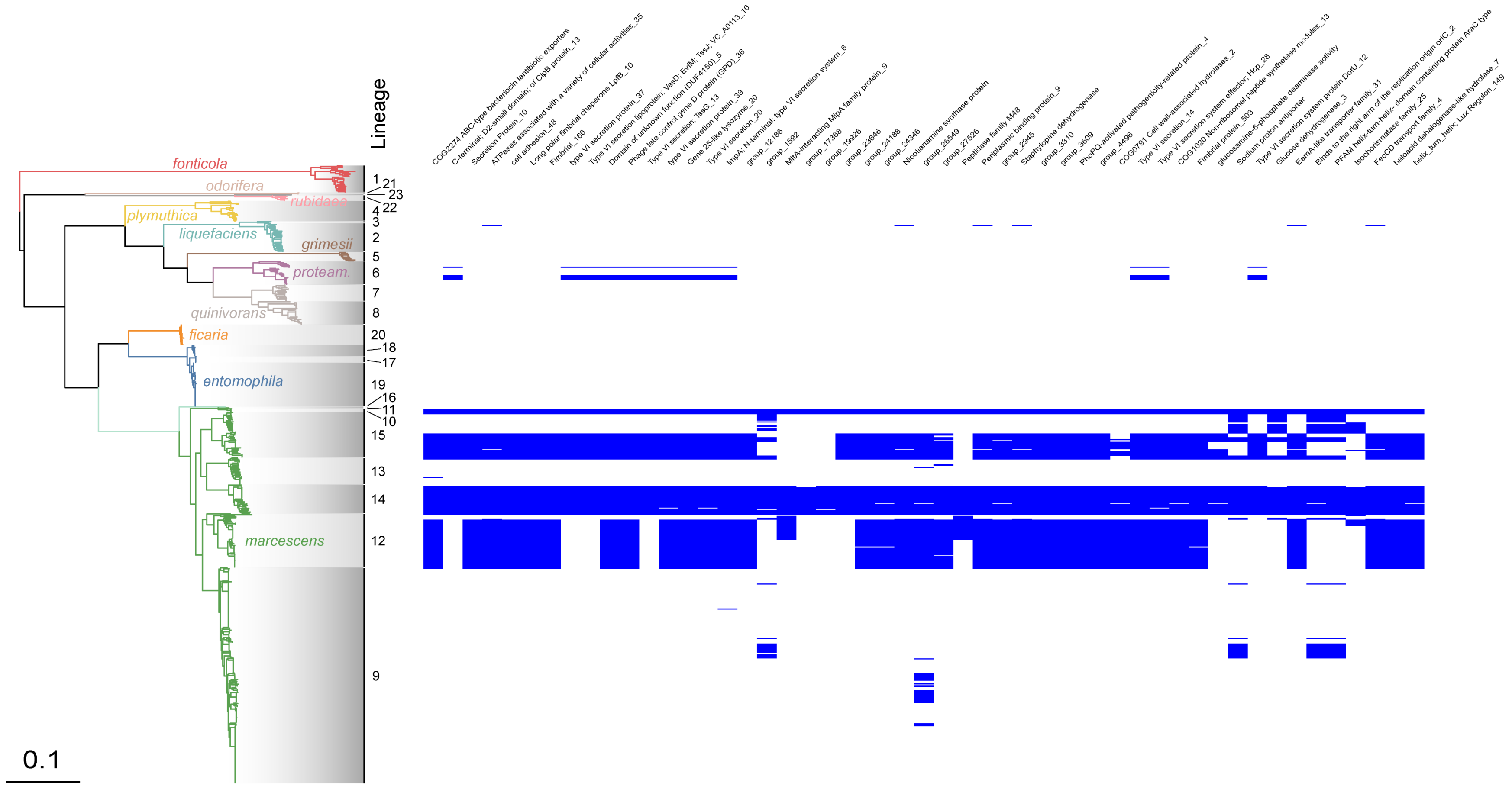

Supplementary Figure 7. Distribution of genes core to *S. marcescens* FastBaps L11, 10 and 14. Intersection of genes which are only core to lineages 11, 10 and 14, extracted and plotted against the phylogenetic tree of *Serratia*. Intersection is highlighted on Fig. 2b.

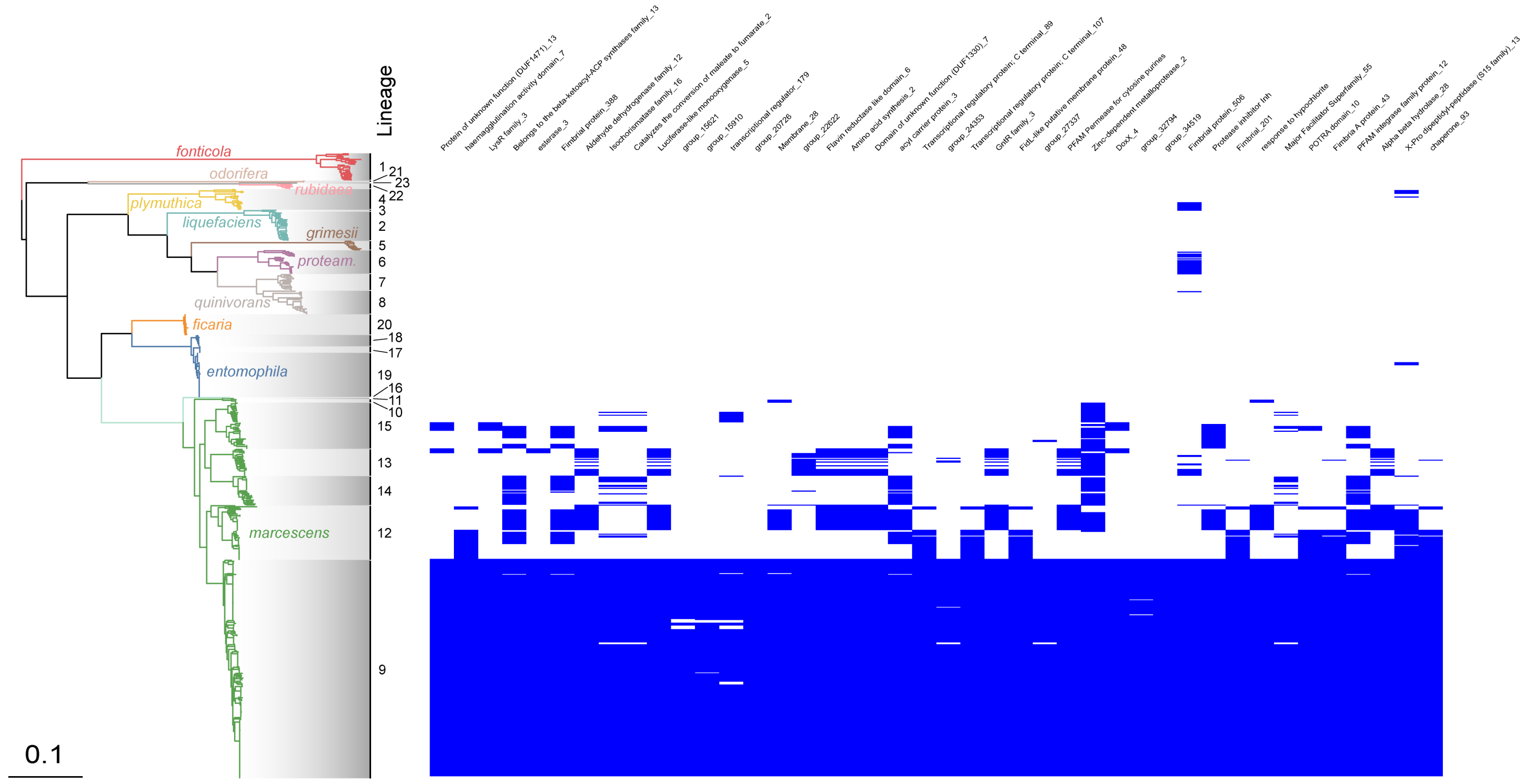

Supplementary Figure 8: Distribution of genes core to *S. marcescens* FastBaps L9. Intersection of genes which are only core to lineage 9, extracted and plotted against the phylogenetic tree of *Serratia*. Intersection is highlighted on Fig. 2b.

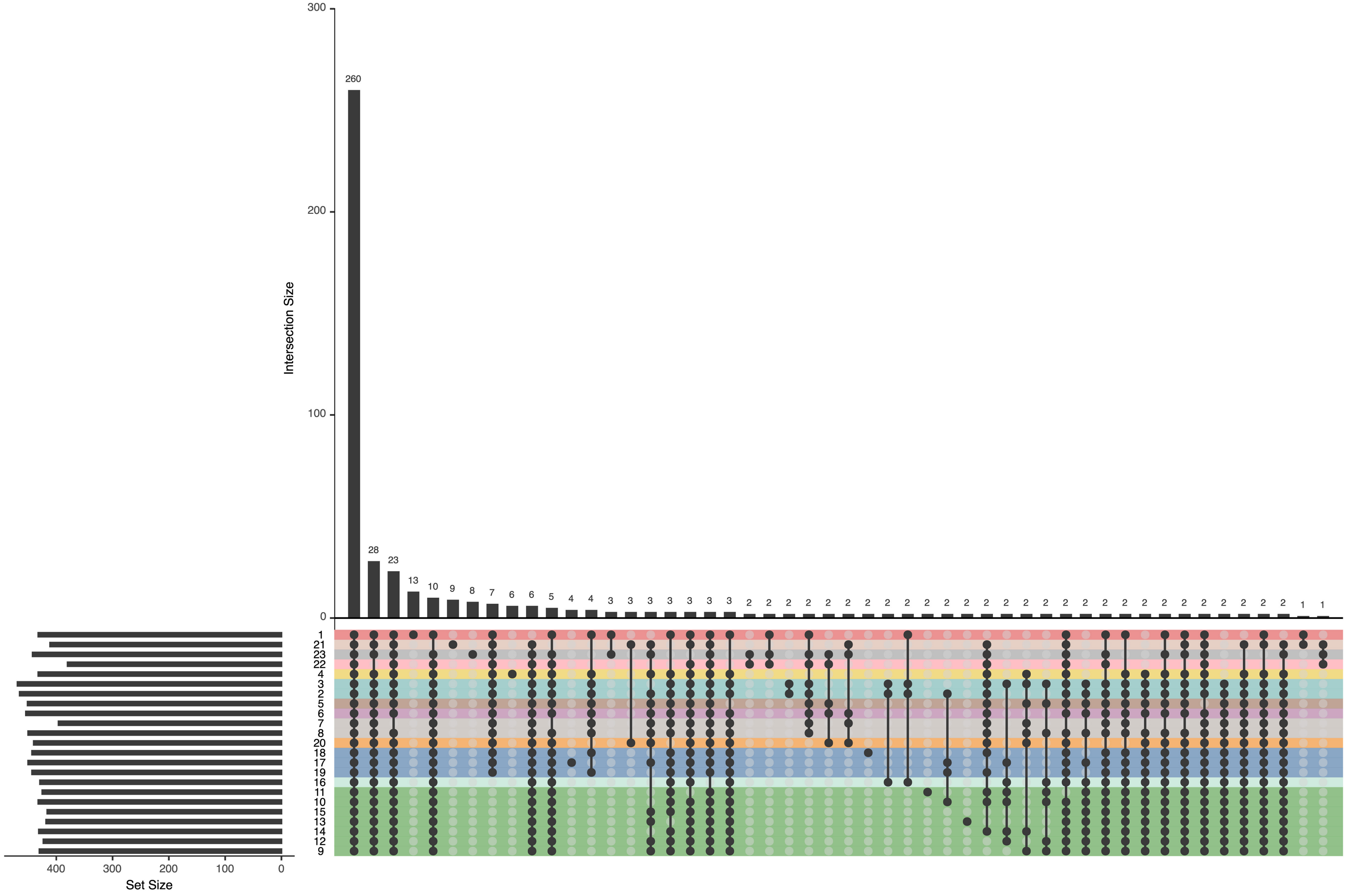

Supplementary Figure 9. Core pathways across *Serratia*. Pathways present in at least 95% of isolates in each FastBaps lineage, presented in an UpSet plot showing the intersections of pathways across FastBaps lineages. Source data are provided as a Source Data file.

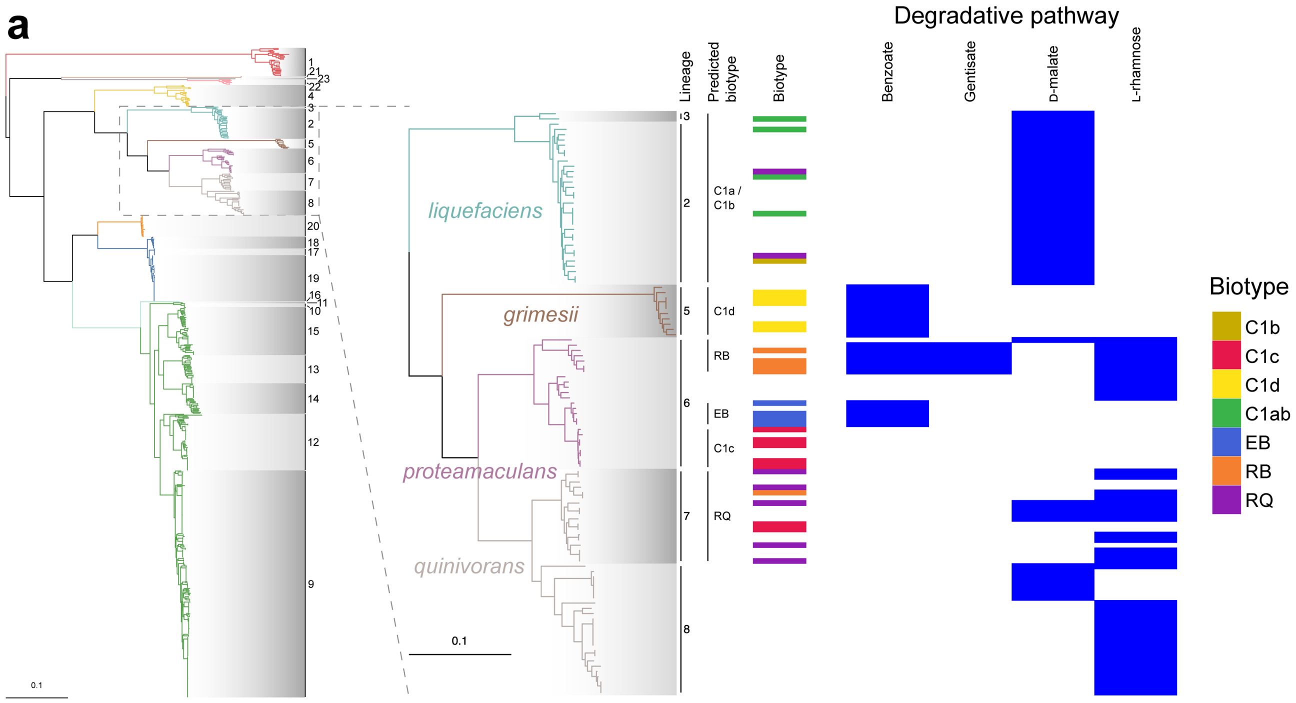

b

	S. liquefaciens	*S. proteamaculans*			*S. quinivorans*	*S. grimesii*	
Biotype (from Grimont & Grimont):	C1ab	C1c	EB	RB	RQ	C1d	ADC
Growth on:							
Benzoate	-	-	(+)	-	-	-	-
Gentisate	-	-	-	+	-	-	-
D-malate	+	-	-	-	v	(v)	(v)
L-rhamnose	-	-	-	+	d	-	-

Supplementary Figure 10. Selected predicted metabolic pathways in the *S. liquefaciens* complex. (a) Presence/absence of selected complete metabolic pathways across the *S. liquefaciens* complex. Pathways selected according to a subset of the biochemical tests used by Grimont and Grimont to group members of the *liquefaciens* complex into species and biotypes. (b) Table of relevant biochemical test results for each biotype, taken from Grimont and Grimont, 2006 (ref 21). Symbols indicate growth phenotype as follows: +, positive for ≥ 90% strains after 2 days; −, negative for ≥ 90% strains after 4 days; v, variable; ( ), 4-day reading; d, indicates test used to differentiate biotypes.

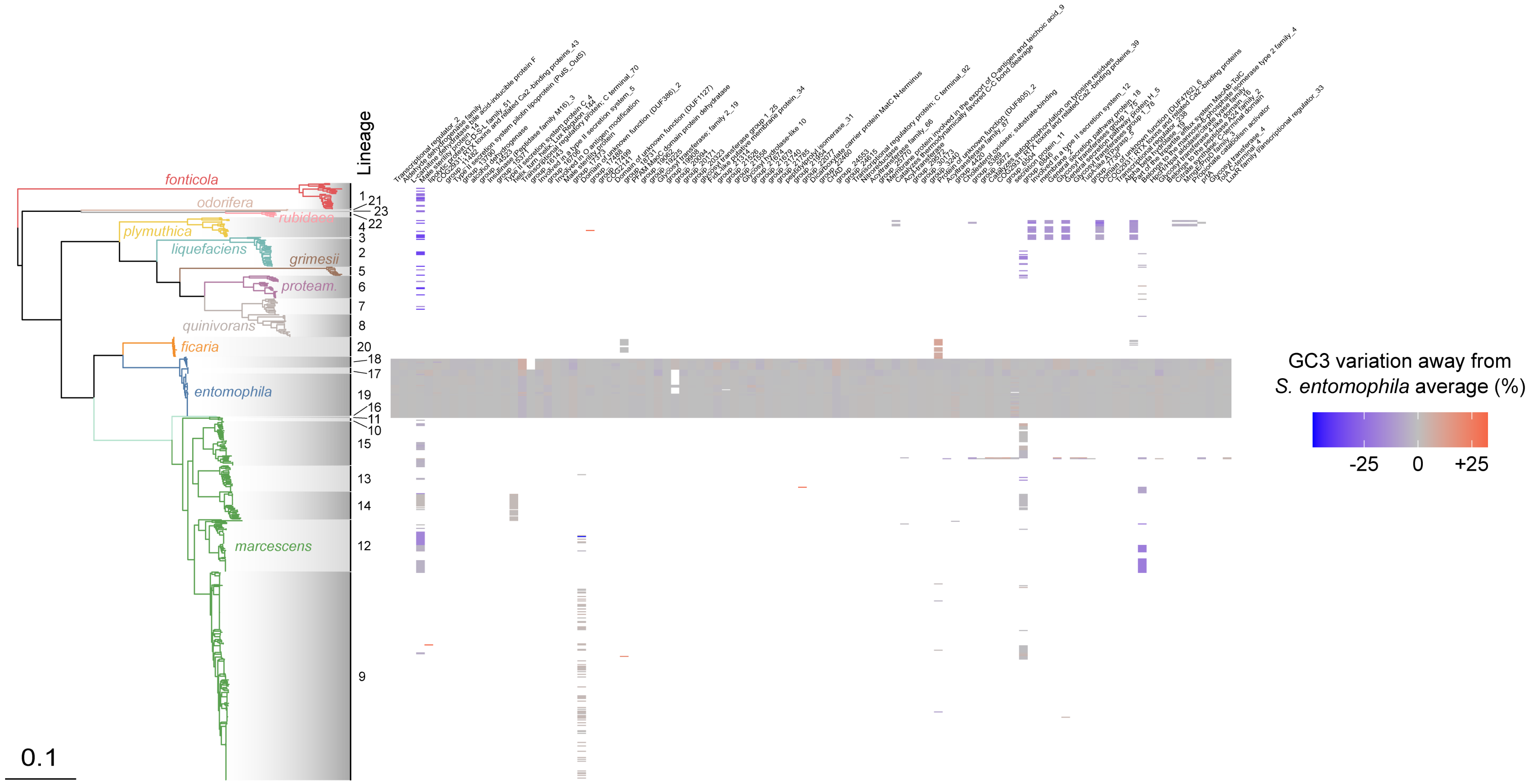

Supplementary Figure 11. GC3 distribution across core genes in *S. entomophila* which are also found elsewhere in the genus. Plot showing the difference between observed GC3 and the mean GC3 across *S. entomophila* (L17-19), calculated separately for each gene group (each column in the heatmap). Colour score corresponds to variation from the per gene group mean GC3 across *S. entomophila* (red = higher, blue = lower). GC3 values for some genes present in Supplementary Fig. 5 are not included here due to the fact that genes are counted as 'present' by Panaroo even when frameshifts and acquired stop mutations are observed. Source data are provided as a Source Data file.

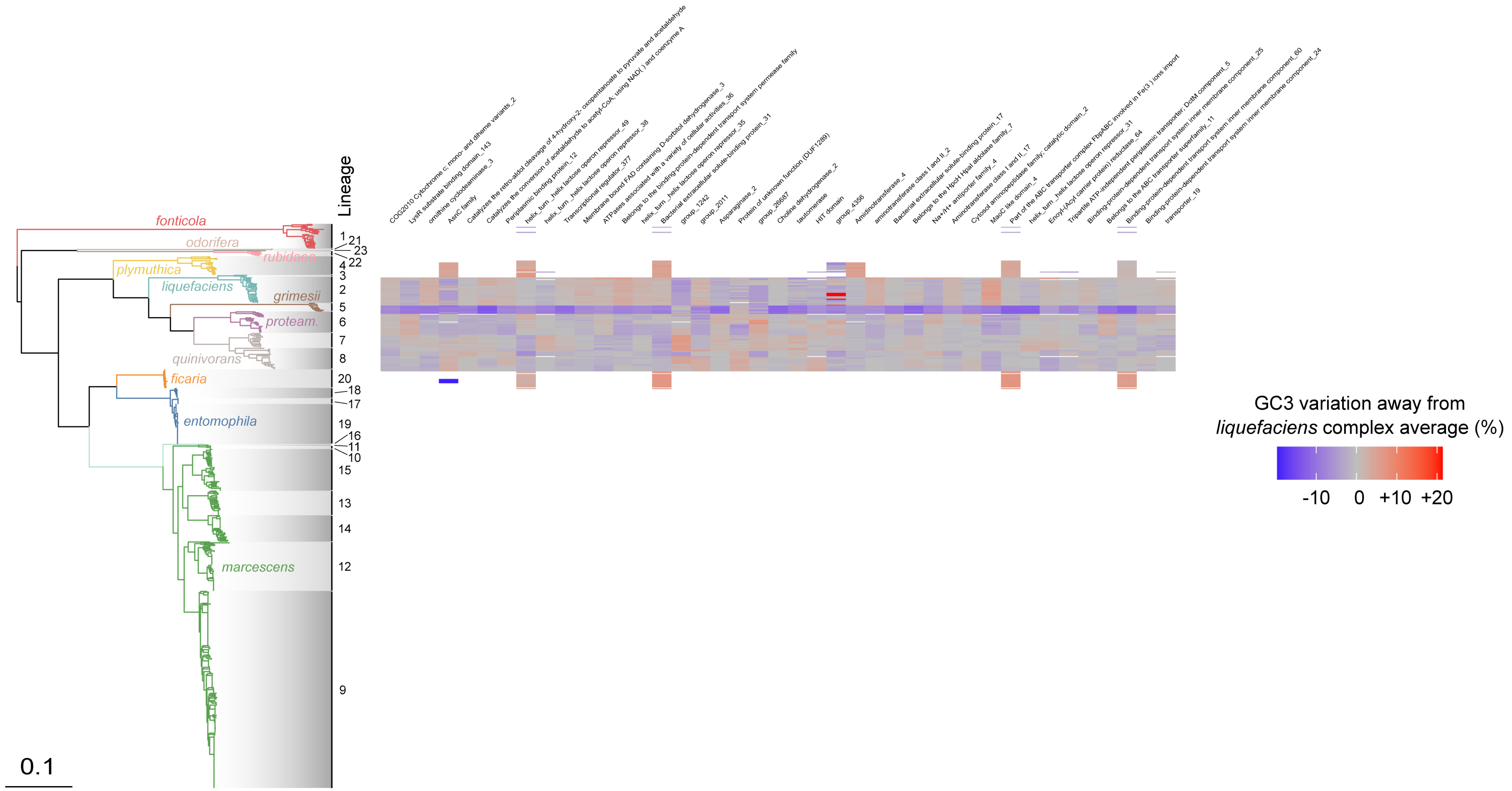

Supplementary Figure 12. GC3 distribution across core genes in the *liquefaciens* complex (*S. liquefaciens, grimesii, proteamaculans, quinivorans*) which are also found elsewhere in the genus. Plot showing the difference between observed GC3 and the mean GC3 across the *liquefaciens* complex (L2, 3, 5-8), calculated separately for each gene group (each column in the heatmap). Colour score corresponds to variation from the per gene group mean across the *liquefaciens* complex (red = higher, blue = lower). GC3 values for some genes present in Supplementary Fig. 6 are not included here due to the fact that genes are counted as 'present' by Panaroo even when frameshifts and acquired stop mutations are observed. Source data are provided as a Source Data file.

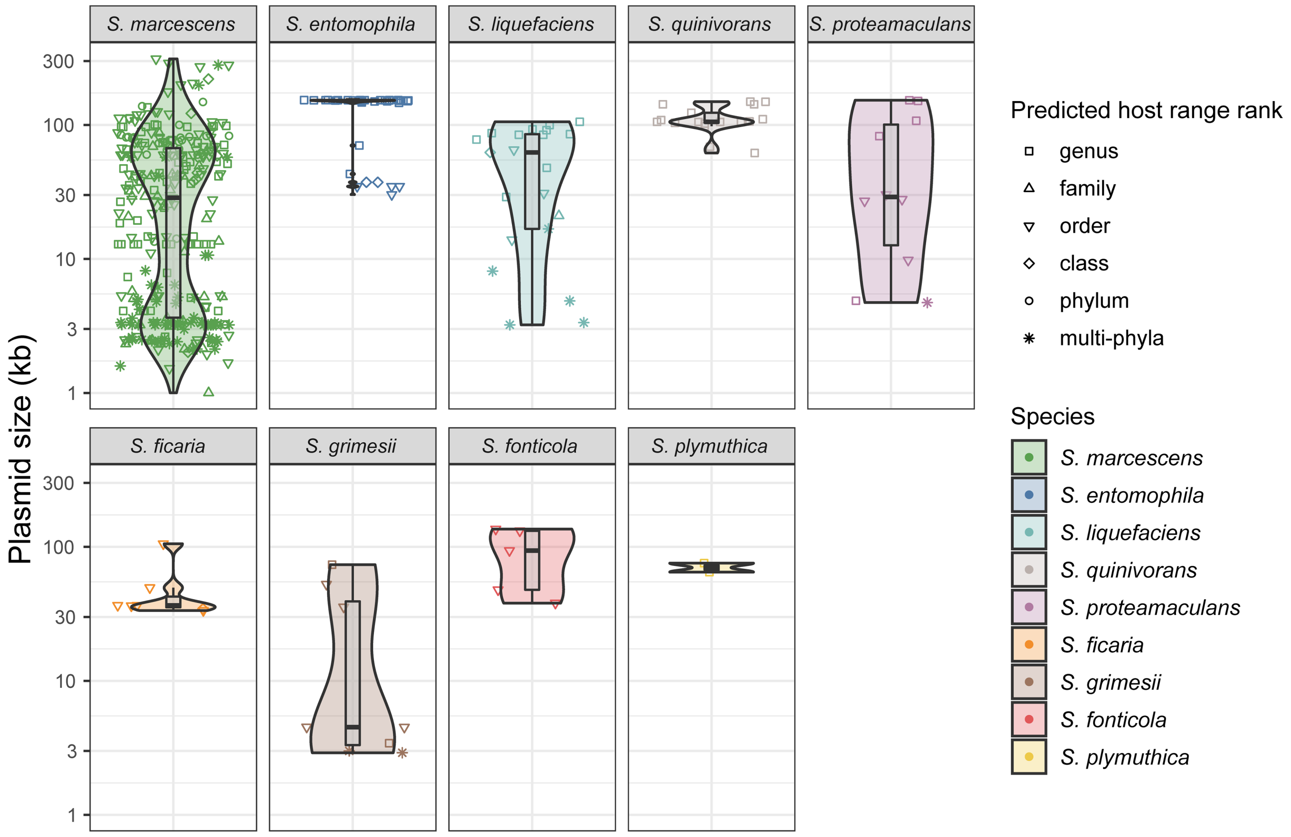

Supplementary Figure 13. Plasmid size distribution per *Serratia* species. Distribution of the size of each predicted plasmid across *Serratia* species, also showing the predicted host range. The predicted host range for each plasmid is represented by the shape of the point. Overlaid violin plots show the overall distribution of plasmid lengths for each species. Boxplots show the median (thick line), first and third quartiles (lower and upper hinges), and whiskers extend to the largest or smallest value within 1.5 times the interquartile range, extended from each higher or lower boxplot hinge (n = 38 plasmids for *S. entomophila*, 7 for *S. ficaria*, 5 for *S. fonticola*, 8 for *S. grimesii*, 21 for *S. liquefaciens*, 301 for *S. marcescens*, 2 for *S. plymuthica*, 10 for *S. proteamaculans*, and 17 for *S. quinivorans*). Source data are provided as a Source Data file.

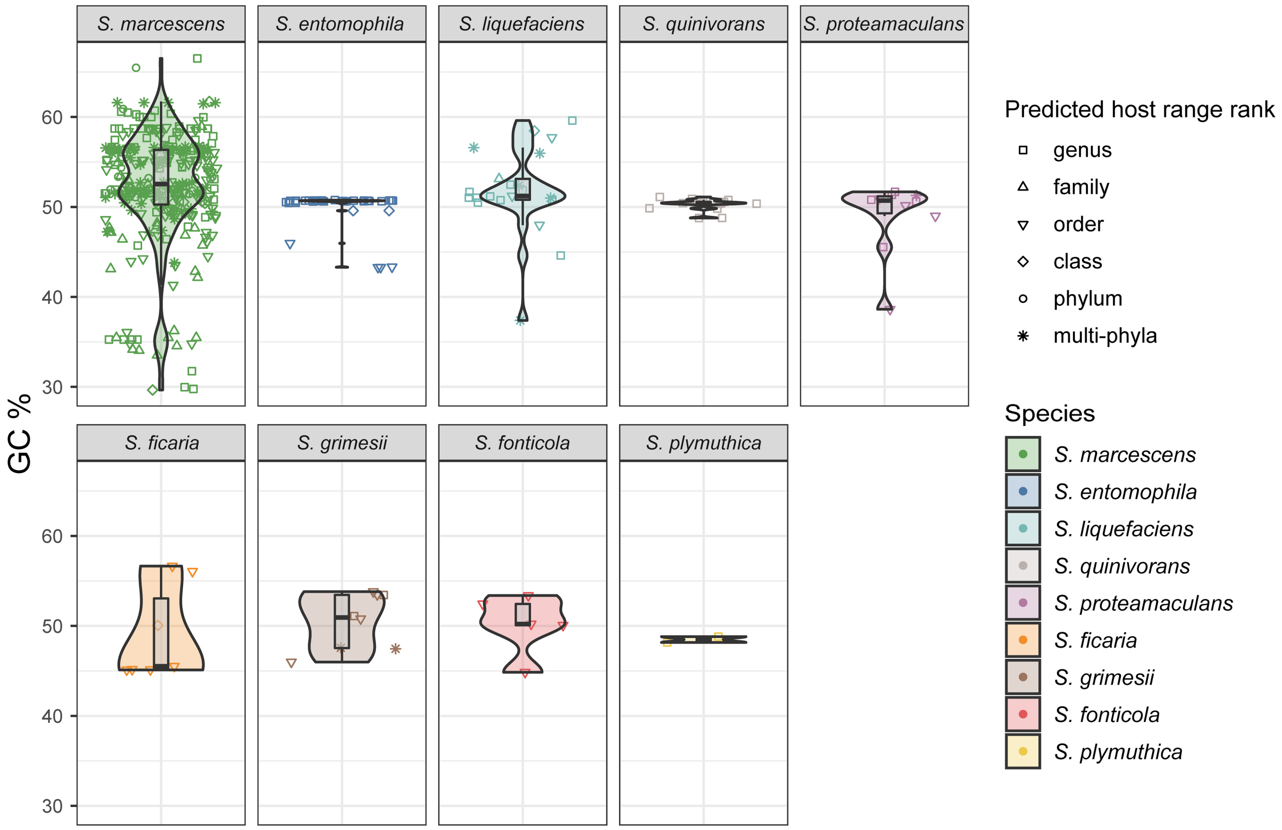

Supplementary Figure 14. Plasmid GC content distribution per *Serratia* species. Distribution of overall GC content for each plasmid across *Serratia* species, also showing the predicted host range. The predicted host range for each plasmid is represented by the shape of the point. Overlaid violin plots show the overall distribution of GC content for plasmids within each species. Boxplots show the median (thick line), first and third quartiles (lower and upper hinges), and whiskers extend to the largest or smallest value within 1.5 times the interquartile range, extended from each higher or lower boxplot hinge (n = 38 plasmids for *S. entomophila*, 7 for *S. ficaria*, 5 for *S. fonticola*, 8 for *S. grimesii*, 21 for *S. liquefaciens*, 301 for *S. marcescens*, 2 for *S. plymuthica*, 10 for *S. proteamaculans*, and 17 for *S. quinivorans*). Source data are provided as a Source Data file.

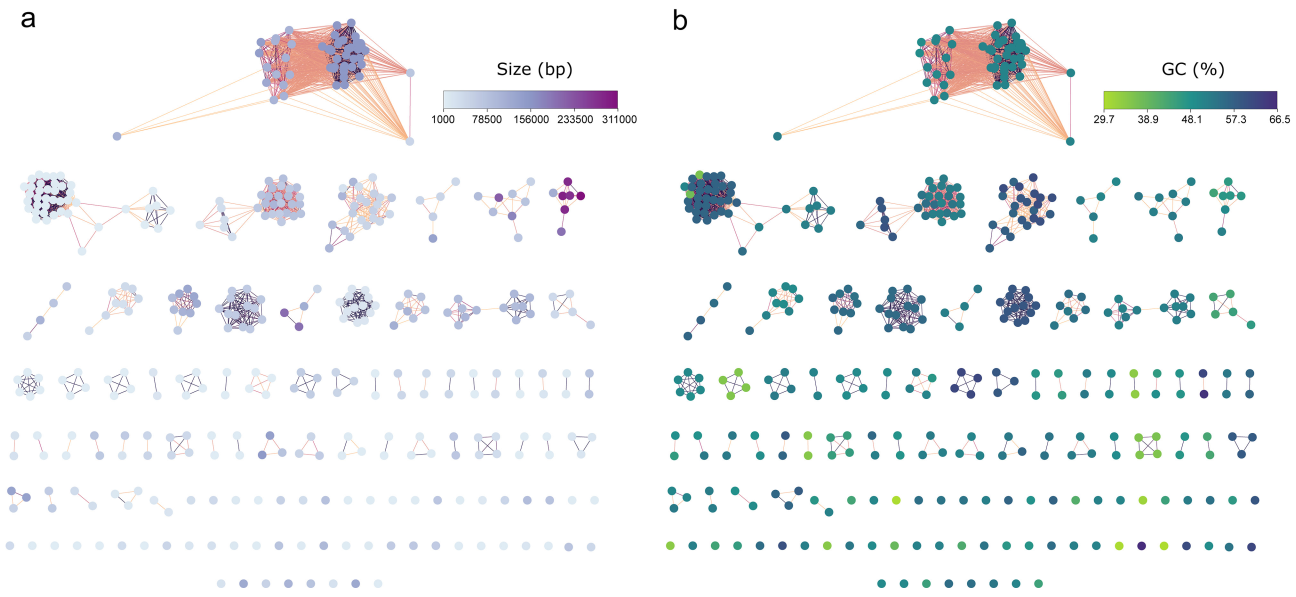

Supplementary Figure 15. Diversity of *Serratia* plasmids according to size and GC content. Plasmid clusters displayed as in Fig. 6 and coloured according to (a) size or (b) GC content.

Supplementary Table 1: Size of core and accessory genomes for species ANI phylogroups. Core genes are classified as any gene present in ≥ 95% of all genomes in each phylogroup. Accessory genes are classified as any gene present in < 95% of all genomes in each phylogroup. Pan-genome analysis was performed using Panaroo on genus-wide set of 664 genomes.

**Species**	**Core**	**Accessory**	**Number of genomes**
S. quinivorans	3921	6419	43
S. marcescens	3697	16351	404
S. fonticola	3797	9159	29
S. liquefaciens	4176	5917	33
S. entomophila	3948	2764	66
S. ficaria	4109	3490	22
S. grimesii	4207	2339	10
S. plymuthica	3732	5445	22
S. proteamaculans	3917	5739	25
S. odorifera	4887	136	2
S. rubidaea	3772	2063	6
S. rubidaea-like	4510	NA	1
S. marcescens-like	4507	NA	1
