# Supplementary material for: The genus Serratia revisited by genomics
Source: Nat Commun. 2022 Sep 3;13:5195. doi: 10.1038/s41467-022-32929-2 (PMC9440931; doi:10.1038/s41467-022-32929-2)
Supplement: Supplementary file 2 — Description of Additional Supplementary Files [file 41467_2022_32929_MOESM2_ESM.pdf]

Title: Supplementary Data 1.
Description: Details of bacterial isolates sequenced and analysed in this study with summaries of sequencing and assembly statistics. Datasets ‘Grimont collection (Institut Pasteur)’ and ‘UK hospitals’ represent those isolates sequenced in this study. Strains highlighted in yellow are type strains of the respective species.

Title: Supplementary Data 2.
Description: Details of plasmid sequences analysed in this study.
