# Supplementary material for: The genus Serratia revisited by genomics
Source: Nat Commun. 2022 Sep 3;13:5195. doi: 10.1038/s41467-022-32929-2 (PMC9440931; doi:10.1038/s41467-022-32929-2)
Supplement: Supplementary file 5 — Reporting Summary [file 41467_2022_32929_MOESM5_ESM.pdf]

nature portfolio

Corresponding author(s): Sarah Coulthurst & Nicholas Thomson

Last updated by author(s): Aug 12, 2022

Reporting Summary

Nature Portfolio wishes to improve the reproducibility of the work that we publish. This form provides structure for consistency and transparency in reporting. For further information on Nature Portfolio policies, see our Editorial Policies and the Editorial Policy Checklist.

Statistics

For all statistical analyses, confirm that the following items are present in the figure legend, table legend, main text, or Methods section.

n/a	Confirmed	
☒	☐	The exact sample size (n) for each experimental group/condition, given as a discrete number and unit of measurement
☒	☐	A statement on whether measurements were taken from distinct samples or whether the same sample was measured repeatedly
☐	☒	The statistical test(s) used AND whether they are one- or two-sided<br>*Only common tests should be described solely by name; describe more complex techniques in the Methods section.*
☒	☐	A description of all covariates tested
☒	☐	A description of any assumptions or corrections, such as tests of normality and adjustment for multiple comparisons
☐	☒	A full description of the statistical parameters including central tendency (e.g. means) or other basic estimates (e.g. regression coefficient) AND variation (e.g. standard deviation) or associated estimates of uncertainty (e.g. confidence intervals)
☐	☒	For null hypothesis testing, the test statistic (e.g. F, t, r) with confidence intervals, effect sizes, degrees of freedom and P value noted<br>*Give P values as exact values whenever suitable.*
☐	☒	For Bayesian analysis, information on the choice of priors and Markov chain Monte Carlo settings
☐	☒	For hierarchical and complex designs, identification of the appropriate level for tests and full reporting of outcomes
☐	☒	Estimates of effect sizes (e.g. Cohen's d, Pearson's r), indicating how they were calculated

Our web collection on statistics for biologists contains articles on many of the points above.

Software and code

Policy information about availability of computer code

Data collection

No software was used for data collection.

Data analysis

Albacore v. 2
Kraken v. 1.1.1
Braken v. 1.0.0
bwa v. 0.7.17
samtools v. 0.1.19
bcftools v. 0.1.19
Quast v. 4.6.0
VelvetOptimiser v. 2.2.5
Velvet v. 1.2
SSPACE
GapFiller
SPAdes v. 3.6.1
Unicycler v. 0.4.7
Canu v. 1.6
Circlator v. 1.5.5
Prokka v.1.13.3
Panaroo v1.2.3
Vegan v. 2.5.7
Twilight (no version number; https://github.com/gh11/twilight)
Roary v. 3.12.0

SNP-sites v2.5.1
IQtree v.1.6.10
networkx package (https://networkx.github.io/)
Cytoscape v. 3.7.1
Fastbaps v. 1.0.4
Pathway tools v. 23.5
Multi-processing wrapper tool mpwt (https://github.com/AuReMe/mpwt)
EggNOG-mapper v. 1.0.3
MOB-suite v. 3.0.3
Mash v. 2.3
Bedtools v2.29.0
hmmer v. 3.2.1
blast+ v.2.2.31
Phaster (https://phaster.ca)
genoplotR v.0.8.11
ggtree v2.4.2
gggenes v. 0.4.1
tidyverse v1.3.1
ggpubr v0.4.0
UpsetR v1.4.0
SSPACE v2.0
Gapfiller v1.11
Clustermaker2 v2.2

Custom scripts are described in the Methods section and are available from the GitHub repository as follows:
https://github.com/djw533/Serratia_genus_paper
https://github.com/djw533/hamburger
https://github.com/djw533/pathwaytolls_gff2gbk
https://github.com/djw533/micro.gen.extra

For manuscripts utilizing custom algorithms or software that are central to the research but not yet described in published literature, software must be made available to editors and reviewers. We strongly encourage code deposition in a community repository (e.g. GitHub). See the Nature Portfolio guidelines for submitting code & software for further information.

Data

Policy information about availability of data

All manuscripts must include a data availability statement. This statement should provide the following information, where applicable:
- Accession codes, unique identifiers, or web links for publicly available datasets
- A description of any restrictions on data availability
- For clinical datasets or third party data, please ensure that the statement adheres to our policy

The sequencing data generated in this study have been deposited in the European Nucleotide archive (ENA; https://www.ebi.ac.uk/ena/), under accession numbers ERP106480 for the Pasteur collection (https://www.ebi.ac.uk/ena/browser/view/PRJEB24638) and ERP135711 for the UK hospitals collection (https://www.ebi.ac.uk/ena/browser/view/PRJEB51113). The annotated genome assemblies for both collections have been deposited in the ENA under the project accession numbers above: ERP106480 for the Pasteur collection (https://www.ebi.ac.uk/ena/browser/view/PRJEB24638) and ERP135711 for the UK hospitals collection (https://www.ebi.ac.uk/ena/browser/view/PRJEB51113). The other whole genome sequences used in this study are available in the NCBI GenBank (https://ftp.ncbi.nlm.nih.gov/genomes/genbank/), with the accession numbers for the individual sequences provided in Supplementary Data 1. The gene annotations generated for all 664 genomes analysed in this study, together with all the genome assemblies generated during this study, are available though Figshare (https://doi.org/10.6084/m9.figshare.18051824). All other data used in figure generation, including the output of pan-genome analysis, are available in the same repository (https://doi.org/10.6084/m9.figshare.18051824). The MiniKraken reference database used for species identification is available at https://ccb.jhu.edu/software/kraken/dl/minikraken_20171019_8GB.tgz and the MOB-suite reference database used for plasmid replicon identification is available from Zenodo (https://doi.org/10.5281/zenodo.3785612). Source data are provided with this paper.

Field-specific reporting

Please select the one below that is the best fit for your research. If you are not sure, read the appropriate sections before making your selection.

☒ Life sciences ☐ Behavioural & social sciences ☐ Ecological, evolutionary & environmental sciences

For a reference copy of the document with all sections, see nature.com/documents/nr-reporting-summary-flat.pdf

Life sciences study design

All studies must disclose on these points even when the disclosure is negative.

Sample size | No sample size calculations were performed. We sequenced as many viable and available bacterial isolates as was practical. Limitations were

imposed by the strains available in the collections and the fact that not all the archived historical cultures were viable. Our study used a larger and more balanced dataset than previous genomics studies involving this genus.

Data exclusions	Duplicate strains, non-Serratia strains or publicly available sequences with poor quality assemblies were excluded, as described in the Methods section:<br><br>Sequence data quality control:<br>Read sets obtained from all samples were compared to the MiniKraken database by Kraken v0.1057, and then corrected using Bracken58 which assigns reads to a specific reference sequence, species or genus. If reads were not able to be assigned to a taxonomic class, they were classed as 'unclassified'. Any read sets that belonged to genera other than Serratia were discarded from any further analysis, along with any assemblies obtained from those read sets.<br>Any read sets with more than an estimated five percent of heterozygous SNPs across the whole genome were removed from further analysis, in addition to any assemblies obtained from those read sets. Heterozygous SNPs were calculated using a software pipeline from the pathogen informatics team at the Wellcome Sanger Institute. Specifically, read sets from each Serratia sample were aligned to the genome of S. marcescens Db11. Reads were aligned using bwa v0.7.1759, and parsed using samtools v0.1.1960 and bcftools v0.1.1960. Reads were considered as heterozygous if there were at least two variants at the same base, both supported by a number of reads that was fewer than 90 percent of the total reads mapped to that site. Read coverage to each strand was considered independently. The minimum total coverage required was 4x, and the minimum total coverage for each strand was 2x. Calculated heterozygous SNP coverage was then predicted by scaling the number of observed heterozygous SNPs against the proportion of the reference that was covered by read mapping.<br>Eight genome sequences from the Pasteur collection dataset and one from the UK hospitals set were removed due to the above criteria. In addition, a number of the isolates resuscitated from the Pasteur collection were duplicate samples of the same strain. After inspection of preliminary phylogenetic trees from core-gene alignments (see below), a further 56 genomes were removed from the Pasteur collection dataset due to being duplicates of the same-named strain.<br><br>Publicly available genome sequences:<br>Previously-published, publicly-available assembled genome sequences were downloaded from the NCBI GenBank database (https://ftp.ncbi.nlm.nih.gov/genomes/genbank/) as of 19/03/2019. Genomes were downloaded if the species was attributed to any of the following: Serratia sp., odorifera, rubidaea, plymuthica, liquefaciens, grimesii, oryzae, proteamaculans, quinivorans, nematodiphila, ficaria, entomophila or marcescens. Assemblies smaller than 4.5 Mbp or larger than 6.5 Mbp were removed from the analysis, along with any assemblies comprised of more than 250 contigs. Quast v4.6.061 was used to extract statistics for genomes and genomic assemblies, specifically whole genome GC content, number of contigs and assembly size. Initial phylogenetic trees with additional non-Serratia reference sequences (Yersinia enterocolitica, Rahnella aquatilis and Dickeya solani) were computed, and genomes detemined by visual inspection as being non-Serratia or close to non-Serratia members of Enterobacteriacaeae were removed from any subsequent analysis. Ten genomes were excluded on this basis, including several so-called Serratia sp. and Serratia oryzae.
Replication	Not applicable. Experimental work was restricted to collection of whole genome sequencing data, where replication is not standard practice. Stringent quality control was applied to the data as described in the Methods section.
Randomization	This study was not randomised. Sequences were allocated into lineages L1-L23 on the basis of their phylogenetic position. This study does not involve human participants or animal models and thus randomisation was not required.
Blinding	There was no blinding in this study. As is normal in bacterial genomics studies of this type, the identity/origin of the sequences was known throughout. Since the sequences were grouped during analysis on the basis of objective criteria such as their phylogenetic position, there was no requirement for blinding to reduce the risk of bias; nor was this a study involving human participants or clinical trials.

Reporting for specific materials, systems and methods

We require information from authors about some types of materials, experimental systems and methods used in many studies. Here, indicate whether each material, system or method listed is relevant to your study. If you are not sure if a list item applies to your research, read the appropriate section before selecting a response.

Materials & experimental systems

n/a	Involved in the study	
☒	☐	Antibodies
☒	☐	Eukaryotic cell lines
☒	☐	Palaeontology and archaeology
☒	☐	Animals and other organisms
☒	☐	Human research participants
☒	☐	Clinical data
☒	☐	Dual use research of concern

Methods

n/a	Involved in the study	
☒	☐	ChIP-seq
☒	☐	Flow cytometry
☒	☐	MRI-based neuroimaging
